# A182 THE IMPACT OF DISEASE STATUS AFTER BIOLOGIC INDUCTION THERAPY ON LONG-TERM OUTCOMES IN PERSONS WITH CROHN’S DISEASE

**DOI:** 10.1093/jcag/gwae059.182

**Published:** 2025-02-10

**Authors:** G S Brar, P Tandon, M Haque, N Butt, M Sanon, D Naessens, E Pone, L Targownik

**Affiliations:** University of Toronto Temerty Faculty of Medicine, Toronto, ON, Canada; University of Toronto Temerty Faculty of Medicine, Toronto, ON, Canada; University of Saskatchewan College of Medicine, Saskatoon, SK, Canada; Sinai Health, Toronto, ON, Canada; Janssen Global Services, Horsham, PA; Janssen Global Services, Horsham, PA; Janssen Inc. Canada, Toronto, ON, Canada; University of Toronto Temerty Faculty of Medicine, Toronto, ON, Canada

## Abstract

NOT PUBLISHED AT AUTHOR’S REQUEST

Please acknowledge all funding agencies listed below

**Funding Agencies**: Study Supported by funding from Janssen

